# Different Cultivation Environments Affect the Yield, Bacterial Community and Metabolites of *Cordyceps cicadae*

**DOI:** 10.3389/fmicb.2021.669785

**Published:** 2021-05-11

**Authors:** Zhaoying Zeng, Dan Mou, Li Luo, Wenlin Zhong, Lin Duan, Xiao Zou

**Affiliations:** ^1^Institute of Fungal Resources, College of Life Sciences, Guizhou University, Guiyang, China; ^2^Key Laboratory of Plant Resource Conservation and Germplasm Innovation in Mountainous Region (Ministry of Education), Guizhou Key Lab of Agro-Bioengineering, College of Life Sciences/Institute of Agro-Bioengineering, Guizhou University, Guiyang, China; ^3^School of Ecology and Environmental Science, Yunnan University, Kunming, China

**Keywords:** *Cordyceps cicadae*, cultivation in natural habitat, artificial cultivation, metabolite, bacterial community composition, PICRUSt

## Abstract

*Cordyceps cicadae* is an entomogenous fungus with important uses in traditional Chinese medicine. However, its wild resources have not met consumers’ demand due to excessive harvesting practices. Artificial cultivation is therefore an important alternative, but research on cultivating *C. cicadae* in natural habitats has not been reported. In this study, we aimed to explore the viability of cultivating *C. cicadae* in a natural habitat, in the soil of *Pinus massoniana* forest. We assessed and compared the yield, metabolite contents and bacterial community composition of *C. cicadae* grown in the *Antheraea pernyi* pupae at different growth stages, and under different cultivation conditions, in the soil of a natural habitat and in sterile glass bottles. Our results showed that cultivating *C. cicadae* in a natural habitat is feasible, with up to 95% of pupae producing *C. cicadae* fruiting bodies. The content of nitrogen compounds (amino acids) in *C. cicadae* cultivated in a natural habitat was significantly higher than in glass bottles, while the yield and carbon compound (mannitol and polysaccharide) and nucleoside (cordycepin and adenosine) contents were lower. Different bacterial genera were enriched in *C. cicadae* at different growth stages and cultivation environments, and these bacterial genera were closely related to metabolites contents during growth. This study demonstrated the viability of a novel cultivation method of *C. cicadae*, which could be used as an alternative to wild stocks of this fungus. These findings provided new insights into the growth mechanism of *C. cicadae* and its interaction with soil microorganisms.

## Introduction

*Cordyceps cicadae* is an entomogenous fungus belonging to the family Claviciptaceae that has been used as a traditional Chinese medicine for over 1600 years (Liu, 2012). It has several host cicadas, such as *Cicada flammata* Distant, *Platypleura kaempferi* Fabricius, *Cryptotympana pustulata* Fabricius, *Platylomia pieli* Kato, and *Oncotympana maculatieollis* Motsch (Zeng et al., 2014, 2017), and can form fruiting bodies on the surface of such cicada nymphs after they have been parasitized and killed by *Isaria cicadae*, the anamorphic form of this fungus (Cheng et al., 2006). *C. cicadae* mainly grows in the soil of bamboo, broad-leaved and evergreen broad-leaved forests, but also grows in mixed coniferous and broad-leaved forests. It is geographically widely distributed in provinces south of the Qinling-Huaihe River in China, including Guizhou, Yunnan, Sichuan, and Zhejiang, as well as in Japan, South Korea, Australia, Brazil, and other countries (Liu, 2012; Chen Z.A. et al., 2014; Zeng et al., 2015).

In China, *C. cicadae* is used to treat chronic kidney diseases, palpitations and children with seizures, and is also used as a food and tonic (Hsu et al., 2015; Li et al., 2019). Recent studies have found that *C. cicadae* contains metabolites with important pharmacological functions. Examples of these include the carbon compound mannitol, which is a diuretic, exhibits anti-free-radical activity, and improves cerebral microcirculation and blood flow (Lin et al., 2016; Zhang et al., 2019); polysaccharides, which are also essential carbon compounds, have antioxidant properties and play a role in treating diabetes and improving immune regulation (Lu et al., 2016; Zhang et al., 2018; Wang et al., 2019); adenosine, a nucleoside with anti-inflammatory, neuroprotective and anti-convulsive properties, which has been shown to improve cell viability and prevent and treat neurodegenerative diseases (Latini and Pedata, 2001; Nakav et al., 2008; Olatunji et al., 2016b); and cordycepin, another nucleoside with anti-cancer, immune regulation, and antioxidant functions (Olatunji et al., 2016a; Zhang et al., 2019). Owing to its use as a medicine and tonic, wild *C. cicadae* has been harvested at unsustainable levels, resulting in a significant population reduction in its natural habitats (Liu et al., 2008). The demand for *C. cicadae* cannot be met using these practices, and artificial cultivation is necessary to be an alternative to wild *C. cicadae* and ensure an adequate supply (Hsu et al., 2015; He W.W. et al., 2019; Sun et al., 2019; Nxumalo et al., 2020).

Artificial cultivation techniques for *C. cicadae* include liquid fermentation (Cheng et al., 2006, 2012) and cultivation on cereal medium (Feng, 2002; Zhang et al., 2012; Ke and Lee, 2016) or pupae (Hu et al., 2009; He Y.Q. et al., 2019). Hyphae can only be produced by liquid fermentation, and fruiting bodies only by cereal medium. Cultivation on pupae has become more popular in recent years, because *C. cicadae* with insects and fruiting bodies can be harvested by this way (He W.W. et al., 2019). However, the cultivation of *C. cicadae* on pupae has mainly been conducted in sterile environments, such as sterilized glass bottles, sand, or soil (Hu et al., 2009; Liu et al., 2018); little research has been conducted on cultivating *C. cicadae* in natural habitats, despite a preference among consumers for more “natural” production methods (Huang, 2008).

Wild *C. cicadae* grows in complex natural habitat that are rich in vegetation including *Camellia japonica*, *Camellia oleifera*, *Pyrus pashia*, and *Myrica nana* (Liu, 2012; Zeng et al., 2015). Researches have shown that the habitat soil, sclerotia, and external mycelial cortices of *C. cicadae* enrich abundant microbial communities and share some of the same bacterial genus, and some ectomycorrhizal fungi have also been detected in sclerotia (Zeng et al., 2019; Mou et al., 2021). These results suggested that wild *C. cicadae* populations are ecologically linked to the surrounding plants and soil through microorganisms, which may impact *C. cicadae* growth and production of metabolites. Previous research found that the composition of microbial communities in host varies between different growth environments (Mueller et al., 2020), and changes in microbial community composition in *Cordyceps* spp. can affect growth and metabolite production of host (Zhang et al., 2010; Qu et al., 2019a, b). Cultivating *C. cicadae* in natural habitats, rather than under sterile conditions, may therefore be a helpful experimental approach to exploring soil microbial interactions and their potential impact on growth and metabolite production in *C. cicadae*. In addition, we previously demonstrated that the relative abundance of fungi other than *Isaria* in the sclerotia of *C. cicadae* was less than 1%, while the bacterial community was more abundant and stable (Zeng et al., 2019; Mou et al., 2021). Given their relative abundance compared with fungi, exploring the composition of bacterial communities in the sclerotia of *C. cicadae* is a specific priority in this study.

This study therefore aimed to explore the feasibility of cultivating *C. cicadae* in natural habitats, the bacterial community composition of *C. cicadae* in different cultivation environments, and the relationship between bacterial communities in sclerotia of *C. cicadae* and metabolite production in this medicinally important fungal species. To achieve this, we compared the yield, bacterial community composition and metabolites content of *C. cicadae* cultivated in aseptic glass bottles versus in the soil of a natural *Pinus massoniana* forest habitat.

## Materials and Methods

### Cultivation of *Cordyceps cicadae* in Two Cultivation Environments

The *Isaria cicadae* strain GZUIFR_DJS1 (preserved in Institute of Fungal Resources of Guizhou University) was activated and purified on potato dextrose agar (PDA, 200 g potato extract, 20 g glucose, 20 g agar, 1,000 mL distilled water) solid growth medium. Purified colonies were inoculated in the center of the PDA medium and cultured upside down at 25°C for 5–7 days, until they produced spores. Spore suspensions [concentration (*C*) = 5 × 10^7^ spores/mL] were prepared with sterile ultrapure water containing 0.05% Tween 80 viscous liquid.

**FIGURE 1:**
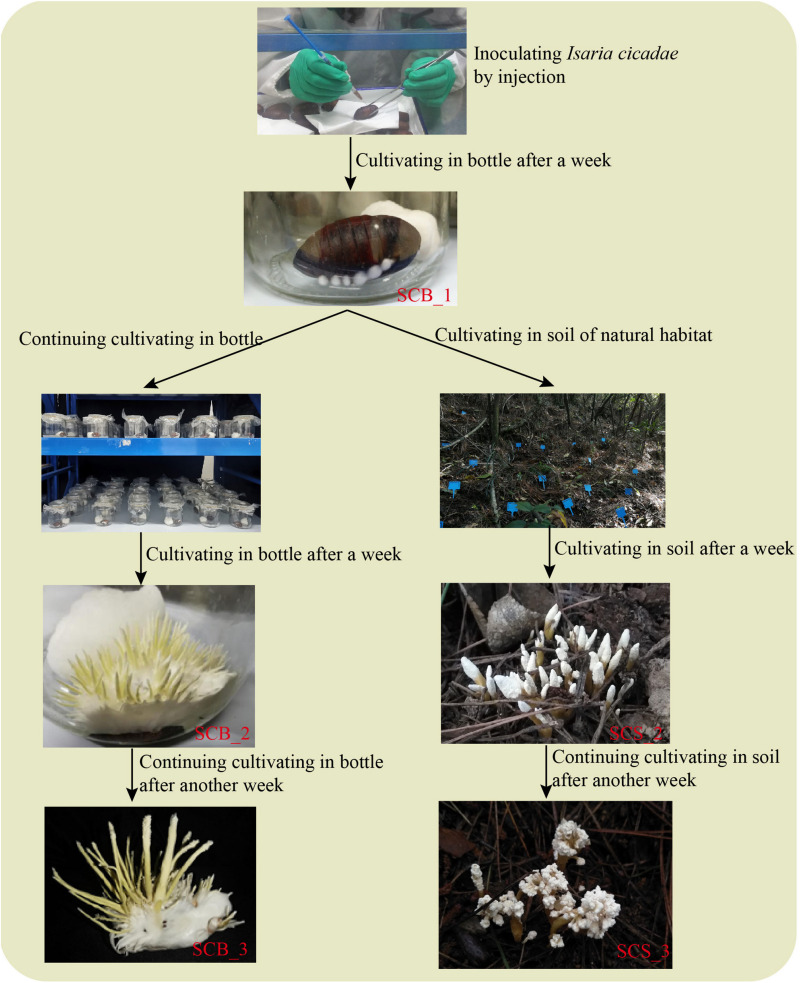
A comparison of *Cordyceps cicadae* cultivation in sterile glass bottles and in the soil of natural habitats.

Pupae of *Antheraea pernyi* Guérin-Méneville, sourced from the Yuxi City Artificial Sericulture Base (Liaoning, China), were selected as host insects (cultivation medium) for *C. cicadae*. Pupae were soaked and scrubbed with 75% alcohol for 10 s for body surface sterilization; pupae were rinsed three times with sterile water to remove any residual alcohol, and then dried with sterile tissue. An inoculation of 0.02 mL spore suspension was made into each pupa at the intersection of the wing and the third somite (Zhang et al., 2016). Inoculated pupae were cultivated in sterile glass bottles at 25°C, each bottle contained a pupa and added a sterile moist cotton ball for maintaining humidity. After a week, the pupae were completely infected by *I. cicadae* and had become mummified. Each pupa was given one of two possible treatments for 2 weeks (Figure 1): either their cultivation in glass bottles was continued (hereafter referred to as CB), or they were cultivated in the soil of natural habitat under a *Pinus massoniana* forest (CS). The experimental cultivation site for natural habitat used in this study was located in the West Campus of Guizhou University (Guizhou, China) and measured approximately 100 m^2^; a detailed description of the habitat is in Supplementary Table 1. Mummified pupae were cultivated in natural habitat under a layer of covering soil approximately 1.5 cm thick, and an interval between any two pupae of 20 cm or more.

### Experiment 1: Effects of Two Cultivation Environments on the Yield of *C. cicadae*

In April 2019, 150 pupae were inoculated and cultivated in sterile glass bottles for 1 week. After removing non-mummified pupae, 120 mummified pupae were randomly divided into six groups of 20; three groups in CB and three in CS. Under both treatment conditions for 2 weeks, *C*. *cicadae* fruiting bodies did not grow anymore and began to produce spores, at this point, *C*. *cicadae* were considered mature; then we harvested all *C*. *cicadae* and washed any soil from CS pupae using tap water. Then, the proportion of pupae producing *C. cicadae* fruiting bodies (i.e., the number of pupae producing fruiting bodies divided by the total number) was calculated. The average proportion of pupae with fruiting bodies across all three groups was then calculated for CB and CS separately. After removing any non-mummified pupae and any *C. cicadae* damaged in the process of washing soil from CS pupae, the fresh weight, fruiting body number, length and diameter of 53 out of 55 *C. cicadae* in CB and 39 out of 57 in CS were measured. Finally, we compared the relative yield between in CB and CS by 100 cicadas i.e., 100 × the proportion of pupae producing *C. cicadae* fruiting bodies × mean fresh weight per *C. cicadae*).

### Experiment 2: Effects of Two Cultivation Environments on the Bacterial Community and Metabolites of *C. cicadae*

In September 2019, 250 pupae were inoculated and cultivated in sterile glass bottles for 1 week. After removing non-mummified pupae, 200 mummified pupae were randomly divided into two groups and cultivated for 2 weeks under CB and CS treatment conditions, respectively. Since the pupae were inoculated with spore suspension, the sclerotia of nine randomly selected pupae were collected for bacterial community analysis each week, with three pupae combined in each of three replicates. Only pupae in the CB treatment condition were taken in week 1; in subsequent weeks, pupae in both CB and CS treatment conditions were taken. Samples were coded according to the week they were taken, e.g., samples from the second week were SCB_2 and SCS_2. Any soil on the CS pupae was washed off with tap water before the external mycelial layer and fruiting body were stripped off, leaving only the mummified pupae. These pupae were soaked in 75% alcohol for 2 min, then soaked in 2.5% sodium hypochlorite solution for 20 s, washed with sterile water five times, and dried with sterile tissue (Xia et al., 2015). Each pupa was crosscut in half on a clean laboratory bench, and the *C. cicadae* sclerotia were picked out with an inoculation needle and placed in sterile centrifuge tubes. Each sample was flash-frozen with liquid nitrogen, ground, and stored at −80°C until the DNA could be extracted. In the third week, the remaining *C. cicadae* in the two treatments were harvested, washed to remove soil, and then dried by dry oven at 80°C. Fruiting bodies were collected, ground and sifted through a 100 mesh screen, and used to detect the contents of five metabolites in *C. cicadae*.

#### Detection of Metabolites

In this study, we measured the content of adenosine, cordycepin, polysaccharides, and mannitol in *C. cicadae* and the content and type of amino acids in *C. cicadae* fruiting bodies. Amino acids were determined using the Hitachi L-8800 high speed amino acid analyzer, according to the national standard protocol *GB 5009.124-2016*. Mannitol content was determined by colorimetry according to a previously established method (San et al., 2010): 50% ethanol was used to extract mannitol using ultrasonic method, then sodium periodate solution, L-rhamnose solution and Nash reagent were used to perform the coloration reaction; absorption values were then detected at 412 nm using an ultraviolet spectrophotometer. Polysaccharide content was determined using a sulfuric acid–phenol method according to the national agricultural standard *NY/T 1676-2008*. Polysaccharides were precipitated in ethanol, and the resulting furfural derivatives of the polysaccharides were dehydrated in concentrated sulfuric acid and condensed with phenol, forming orange-red compounds with a color intensity proportional to the concentration of polysaccharides in the solution. Colorimetric quantification was carried out at 490 nm by an ultraviolet spectrophotometer. Cordycepin and adenosine were determined using high performance liquid chromatography according to national agricultural standard *NY/T 2116-2012*. The chromatographic column used in this study was the Shim-pack VP-ODS 5 micron 250 mm × 4.6 mm. The mobile phase was water and acetonitrile with 95:5 volume (v/v) ratio at a mobile speed of 1.0 mL/min. The temperature of chromatographic column was maintained at 35°C. The contents of cordycepin and adenosine were measured using a UV detector at 260 nm. We conducted three replicates for the measurement of each metabolite.

#### Bacterial DNA Extraction, PCR Amplification, and Sequencing

Bacterial DNA was extracted using the E.Z.N.A.^®^ Soil DNA Kit (Omega Bio-tek), and the concentration and purity of DNA were assessed by electrophoresis on 1.0% weight/volume agarose gels. The primers used to amplify the V4 variable region of the bacterial 16s RNA gene were: 515F (5′-GTGCCAGCMGCCGCGGTAA-3′) and 806R (5′-GGACTACHVGGGTWTCTAAT-3′) (Zhou et al., 2020). DNA amplification was carried out using the ABI GeneAmp 9700 polymerase chain reaction (PCR) instrument. The PCR parameters were as follows: initial denaturation for 3 min at 95°C; denaturation, annealing and extension at 30 s at 95°C, 30 s at 55°C, and 45 s at 72°C for 30 cycles; then final extension for 10 min at 72°C, then 10°C until the reaction stopped. The amplification products were sent to Shanghai Majorbio Bio-pharm Technology Co., Ltd. (Shanghai, China) for sequencing on the Illumina MiSeq sequencing platform.

#### Bioinformatics and Data Analysis

Fast Length Adjustment of SHort reads (FLASH) and Quantitative Insights Into Microbial Ecology (QIIME) software programs were used to merge and quality-filter raw reads from the original DNA fragments (Caporaso et al., 2010; Magoc and Salzberg, 2011). Effective reads were obtained by using a UCHIME algorithm to identify and remove chimeric sequences. Quality sequences were then clustered into operational taxonomic units (OTUs) with a >97% similarity cutoff value using the UPARSE algorithm (Edgar et al., 2011). The Basic Logical Alignment Search Tool (BLAST) was then used to compare representative sequences for each OTU with sequences in the SILVA database to obtain the taxon of each OTU (Quast et al., 2013).

α-diversity of bacterial communities in each sample was represented by Shannon, Chao1 and Coverage indices, calculated after reads were normalized to the minimum reads (30, 665 reads) in each sample. Phylogenetic Investigation of Communities by Reconstruction of Unobserved States (PICRUSt)^1^ software was used to predict the functional profile of bacterial communities using 16S ribosomal ribonucleic acid (rRNA) marker gene sequences (Langille et al., 2013). We then calculated the abundance of Kyoto Encyclopedia of Genes and Genomes (KEGG) pathways and functions at the OTU level in each sample.

The Kolmogorov–Smirnov tests were used to verify the normality of data. Student’s *t*-tests and non-parametric Mann–Whitney *U*-tests were used to identify differences in yield and abundance of KEGG pathways and functions between CB and CS treatment conditions that the data were normal or not, respectively. One-way analysis of variance (ANOVA) and *post hoc* comparisons were performed using Duncan’s Multiple Range test to compare α-diversity among the different groups. The Spearman’s rank correlation coefficient between contents of metabolites mentioned above and the top 30 most abundant bacterial genera was calculated and then visualized by the “pheatmap” package in the R software program. Visualizations of the yield index, the α-diversity index and the bacterial community composition were produced using GraphPad Prism 7 (GraphPad Software Inc., San Diego, CA, United States).

## Results

### Lower Yield of *C. cicadae* Under Natural Habitat Compared With Sterile Bottle

**FIGURE 2:**
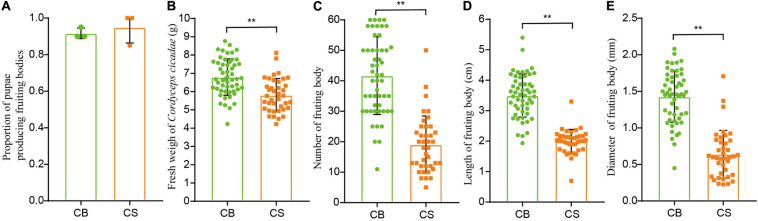
The effects of two cultivation environments on yield measurements in *Cordyceps cicadae*. **(A)** proportion of pupae producing fruiting bodies; **(B)** fresh weight of *Cordyceps cicadae*; **(C)** number of fruiting bodies; **(D)** length of fruiting bodies; **(E)** diameter of fruiting bodies. CB, cultivation in glass bottles; CS, cultivation in the soil of a natural habitat. **p < 0.01, according to non-parametric Mann–Whitney *U*-tests, error bars depict the standard deviation.

The proportion of pupae producing fruiting bodies was similar between CS and CB cultivation environments (92 ± 14% in CS and 95 ± 9% in CB, Figure 2A), indicating that *C. cicadae* can be cultivated in the soil of a natural forest habitat as in glass bottle. However, the fresh weight, number, length and diameter of fruiting bodies in CS were all significantly lower than those in CB (*p* < 0.01, Figures 2B–E), and the relative yield in CS (534.52g/100 cicada) was also lower than that in CB (645.05g/100 cicada, Supplementary Table 2), indicating that the overall growth of *C. cicadae* in CB was greater than in CS.

### Contents of Metabolites in *C. cicadae* Differed Between Two Cultivation Environments

**FIGURE 3:**
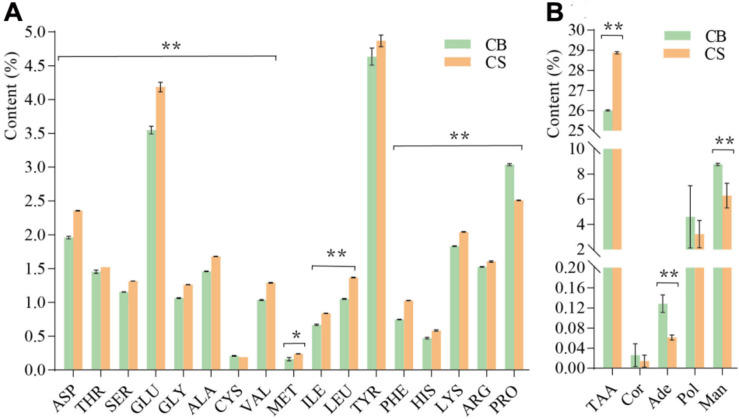
The effects of two cultivation environments on metabolites produced by *Cordyceps cicadae*. **(A)** content of 17 amino acids; **(B)** content of five metabolites. ASP, aspartic acid; THR, threonine; SER, serine; GLU, glutamic acid; GLY, glycine; ALA, alanine; CYS, cysteine; VAL, valine; MET, methionine; ILE, isoleucine; LEU, leucine; TYR, tyrosine; PHE, phenylalanine; HIS, histidine; LYS, lysine; ARG, arginine; PRO, proline; TAA, total amino acid; Cor, cordycepin; Ade, adenosine; Pol, polysaccharide; Man, mannitol. Error bars depict the standard deviation (*p < 0.05, **p < 0.01, according to Student’s *t*-tests).

In total, 17 amino acids were detected in *C. cicadae* cultivated in both environments (Figure 3A). The total amino acid content of *C. cicadae* in CS (28.87 ± 0.057%) was significantly higher than in CB (26.01 ± 0.029%, *p* < 0.01, Figure 3B). The contents of aspartic acid, threonine, serine, glutamic acid, glycine, alanine, cysteine, valine, methionine, isoleucine, phenylalanine, histidine, lysine, arginine, and leucine of *C. cicadae* were significantly higher in CS than in CB (*p* < 0.01). Conversely, the proline content in CS (2.51 ± 0.005%) was significantly lower than that in CB (3.04 ± 0.015%, *p* < 0.01). Cordycepin and polysaccharide contents of *C. cicadae* were slightly lower in CS than in CB. Adenosine and mannitol contents were significantly lower in CS than CB (*p* < 0.01, Figure 3B). These results indicate that cultivating *C. cicadae* in natural habitats increases the production of nitrogen compounds (amino acids), but decreases that of carbon compounds (polysaccharides and mannitol), and nucleosides (cordycepin and adenosine).

### Bacterial Communities in Sclerotia of *C. cicadae* Were Higher Richness When Cultivated in Natural Habitat Compared With Sterile Bottle

A total of 2,931,602 raw sequences were detected. After filtering, 1,465,801 effective reads were obtained, with 30,665–92,371 in each sample. The coverage index of samples (>0.99 for all samples, Supplementary Table 3) and the rarefaction curves (Supplementary Figure 1) indicated that the sampling depth of sequence data set in the current study was adequate for our analyses as the Shannon index flattens out after approximately 30,000 reads in each sample.

**FIGURE 4:**
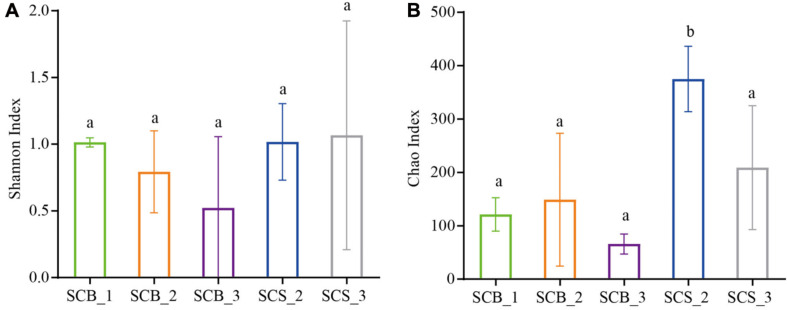
Diversity index of bacterial communities in sclerotia of *Cordyceps cicadae* under the two cultivation environments (SCB, sclerotia of *Cordyceps cicadae* cultivating in glass bottles; SCS, sclerotia of *Cordyceps cicadae* cultivating in the soil of a natural habitat) at 1, 2, and 3 weeks (denoted by _1, _2, and _3, respectively) after inoculation. **(A)** Shannon index of diversity; **(B)** Chao1 index of richness. Different lowercase letters indicate significant difference (*p* < 0.05). Error bars depict the standard deviation.

There was no significant difference in the Shannon diversity index of bacterial communities in the sclerotia of *C. cicadae* in each growth stage between the two environments, indicating that the bacterial community diversity was stable (Figure 4A). However, the Chao1 richness index in CS was higher than in CB; at week 2, the Chao1 richness index was significantly higher in SCS_2 than in SCB_2 (*p* < 0.05, Figure 4B). This result indicates bacterial richness is higher in *C. cicadae* cultivated in natural habitats than sterile bottle.

### Bacterial Community Composition in Sclerotia of *C. cicadae* Varied Between Cultivation Environments and Growth Stages

**FIGURE 5:**
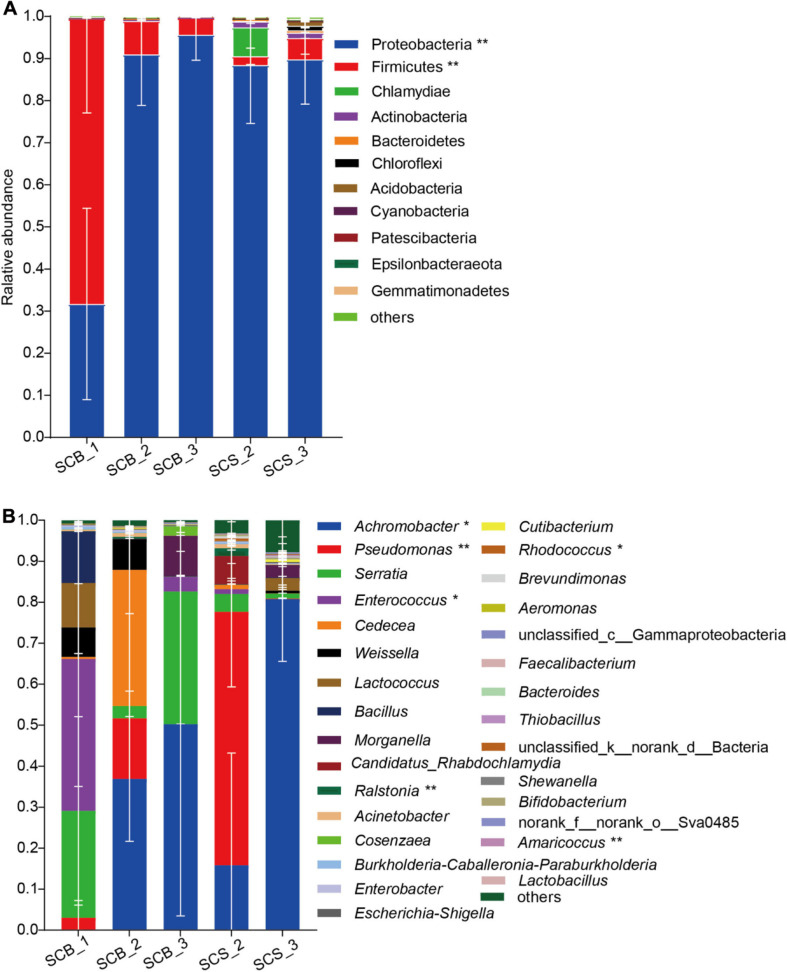
Bacterial community composition in sclerotia of *Cordyceps cicadae* at different growth stage in both cultivation environments (explanations for abbreviations in *X* axis are shown in Figure 4). **(A)** Phyla with relative abundance greater than 0.1%; **(B)** Top 30 most abundant genera (* and ** denotes significance between all growth stages in both environments of *p* < 0.05 and *p* < 0.01, respectively, according to ANOVA, error bars depict the standard deviation).

In total, 11 phyla with a relative abundance greater than 0.1% were detected (Figure 5A). Proteobacteria was the dominant phylum (31.69 ± 22.74%–95.66 ± 6.09%) across all growth stages in both environments. The Firmicutes represented the dominant phylum in SCB_1, and its abundance at this time point in week 1 (67.80 ± 22.2%) was significantly higher than in week 2 or 3 under both cultivation environments (2.08 ± 1.95%–7.93 ± 1.27%, *p* < 0.01).

At the genus level (Figure 5B), the bacterial community composition varied between CB and CS and also across growth stages. In *C. cicadae* cultivated in sterile conditions: *Enterococcus* (37.05 ± 31.08%) and *Serratia* (26.08 ± 22.96%) were the dominant genera in SCB_1; *Achromobacter* (36.91 ± 15.24%) and *Cedecea* (33.18 ± 40.63%) were dominant in SCB_2; and *Achromobacter* (50.24 ± 46.77%) and *Serratia* (32.34 ± 56.01%) were dominant in SCB_3. In *C. cicadae* cultivated in natural habitats, *Pseudomonas* (61.80 ± 18.32%) and *Achromobacter* (80.75 ± 15.18%) were the dominant genera in SCS_2 and SCS_3, respectively. The relative abundance of *Achromobacter* gradually increased from 0.02 ± 0.012% to 80.75 ± 15.18% with the growth of *C. cicadae*, which showed significantly different relative abundance among groups (*p* < 0.05). The relative abundance of *Pseudomonas*, *Enterococcus*, *Ralstonia*, *Rhodococcus*, and *Amaricoccus* were also significantly different among groups. *Pseudomonas*, *Ralstonia*, *Rhodococcus*, and *Amaricoccus* had a significantly higher relative abundance in SCS_2 than at other stages in both environments (*Rhodococcus*, *p* < 0.05; other *p* < 0.01), while *Enterococcus* had significantly higher relative abundance in SCB_1 than at other stages in both environments (*p* < 0.05). *Cedecea* only had higher relative abundance (33.18 ± 23.46%) in SCB_2, but a low relative abundance (0.001 ± 0.00095%–1.09 ± 1.55%) at other stages in both environments (Figure 5B). These data show that the bacterial community composition and dominant genera in the sclerotia of *C. cicadae* vary significantly between cultivation environments and growth stages.

### Predictive Functional Profiling of Bacterial Communities

**FIGURE 6:**
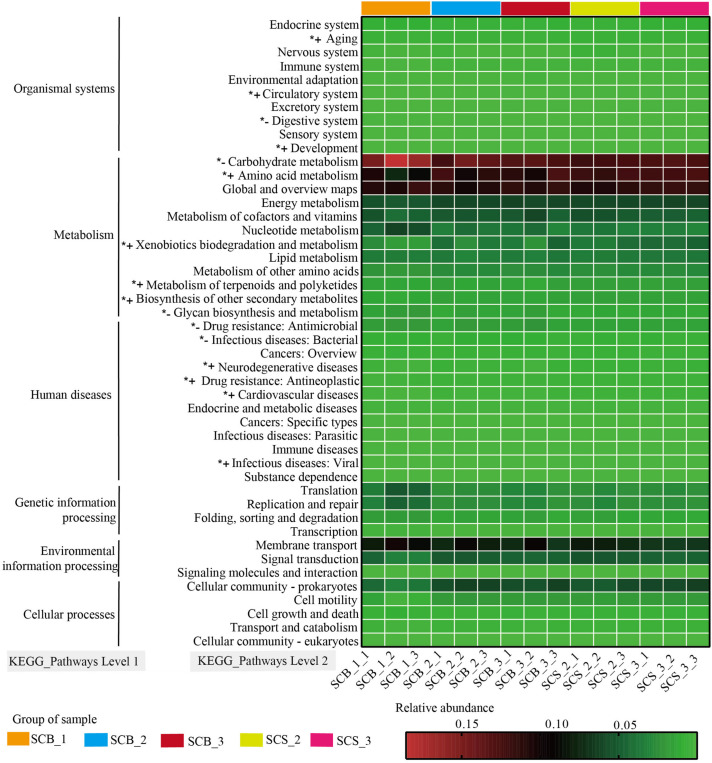
Variation in KEGG metabolic pathways in functional bacterial communities of *Cordyceps cicadae* cultivated in different cultivation environments (SCB, sclerotia of *Cordyceps cicadae* cultivating in glass bottles; SCS, sclerotia of *Cordyceps cicadae* cultivating in the soil of a natural habitat) at 1, 2, and 3 weeks after inoculation (_1, _2, and _3, respectively). The final number in each sample label represents the replicate number of each sample (_1, _2, and _3, respectively). *^+^ denotes the relative abundance of bacteria related to the function is significantly higher in CS than that in CB, *^–^ represents the relative abundance of bacteria related to the function significantly lower in CS than that in CB (*p* < 0.05, according to Student’s *t*-test).

The functions of bacterial communities were predicted by PICRUSt to determine the changes in relative abundance of bacterial OTUs related to functions in samples from different cultivation environments and time points (Figure 6). Bacteria OTUs related to metabolism had the highest relative abundance (62.80–66.33%) at the KEGG pathway Level 1, followed by environmental information processing (11.10–14.06%), genetic information processing (6.04–12.14%), human diseases (4.59–5.29%), cellular processes (2.99–4.04%), and organismal systems (1.71–2.36%).

At the KEGG pathway Level 2, bacteria OTUs with function related to carbohydrate metabolism (11.98–18.53%), global and overview maps (10.10–11.81%), Amino acid metabolism (7.47–12.58%) in metabolism of Level 1 and membrane transport (6.55–10.11%) in environmental information processing of Level 1 showed higher relative abundance than other functions. The relative abundance of bacteria OTUs related to glycan biosynthesis and metabolism and carbohydrate metabolism were significantly lower in CS (1.26 ± 0.06% and 12.42 ± 0.31%, respectively) than in CB (1.57 ± 0.29% and 14.28 ± 2.04%, respectively; both *p* < 0.05). Conversely, in CS compared with CB, the relative abundance of bacteria OTUs related to amino acid metabolism (12.08 ± 0.39% versus 10.44 ± 1.59%), xenobiotic biodegradation and metabolism (4.12 ± 0.50% versus 3.04 ± 1.02%), metabolism of terpenoids and polyketides (1.67 ± 0.03% versus 1.46 ± 0.15%), and biosynthesis of other secondary metabolites (1.60 ± 0.04% versus 1.42 ± 0.13%) were all significantly higher (all *p* < 0.05). These results suggested the relative abundance of bacteria OTUs with functions related to metabolism were significantly different between two cultivation environments, and further indicated the potential correlation between the changes of metabolite content and bacterial community composition in *C. cicadae* cultivated in different cultivation environments.

### The Relationship Between Bacterial Community and Metabolite

**FIGURE 7:**
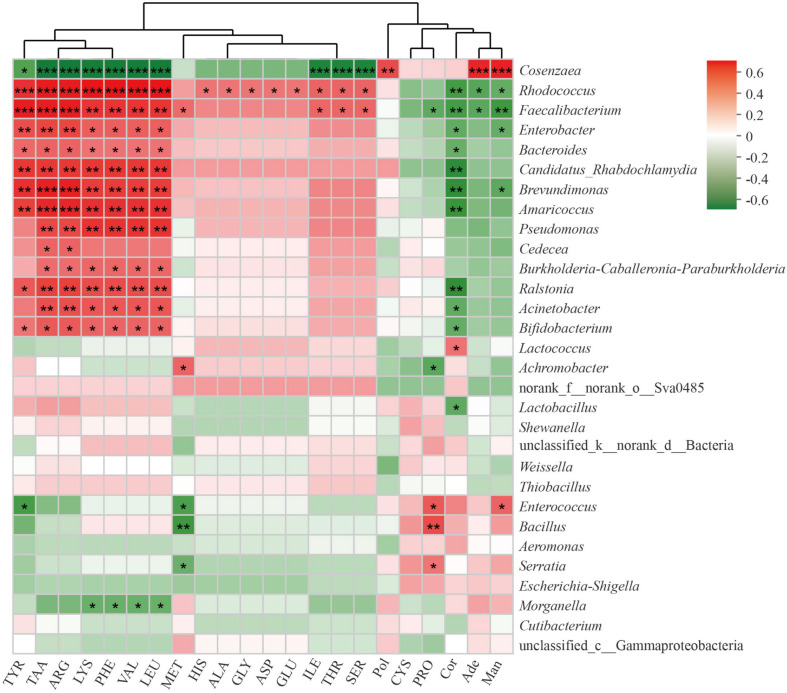
Correlation of top 30 most abundant bacteria genera with metabolites in *Cordyceps cicadae.* The strength (Spearman’s ρ value) of correlations are shown here as different shades of red (positive correlation) and green (negative correlation). Explanations for abbreviations in *X* axis are shown in Figure 3. *0.01 < *p* ≤ 0.05; **0.001 < *p* ≤ 0.01; ***p ≤ 0.001.

To further understand the relationship between bacterial communities and metabolites contents in cultivated *C. cicadae*, we calculated the Spearman’s rank correlation coefficient between the top 30 most abundant genera and the contents of 22 metabolites, including 17 amino acids (Figure 7). There was a significant correlation between most bacterial genera and metabolite contents, especially those of amino acids.

Total amino acids, tyrosine, arginine, lysine, phenylalanine, valine, and leucine were all significantly positively correlated with *Rhodococcus*, *Faecalibacterium*, *Enterobacter*, *Bacteroides*, *Candidatus Rhabdochlamydia*, *Brevundimonas*, *Amaricoccus*, *Ralstonia*, and *Bifidobacterium*, but negatively correlated with *Cosenzaea* (all *p* < 0.05 at least). *Pseudomonas*, *Burkholderia-Caballeronia-Paraburkholderia*, and *Acinetobacter* were all significantly correlated with total amino acids, arginine, lysine, phenylalanine, valine and leucine (all *p* < 0.05 at least). Proline were significantly positively correlated with *Serratia*, *Bacillus*, and *Enterococcus*, but negatively correlated with *Achromobacter* and *Faecalibacterium* (all *p* < 0.05 at least). There was a significant positive correlation between polysaccharide and *Cosenzaea* (*p* < 0.01). Cordycepin was significantly negatively correlated with numerous genera, including *Rhodococcus*, *Faecalibacterium*, *Enterobacter*, *Bacteroides*, *Candidatus_Rhabdochlamydia*, *Brevundimonas*, *Amaricoccus*, *Ralstonia*, *Acinetobacter*, *Bifidobacterium*, and *Lactobacillus*, but significantly positively correlated with *Lactococcus* (all *p* < 0.05 at least). Adenosine were significantly positively correlated with *Cosenzaea* (*p* < 0.01), but negatively correlated with *Rhodococcus* and *Faecalibacterium* (both *p* < 0.05). Mannitol were significantly positively correlated with *Cosenzaea* and *Enterococcus*, but significantly negatively correlated with *Rhodococcus*, *Faecalibacterium*, *Enterobacter*, and *Brevundimonas* (all *p* < 0.05 at least).

## Discussion

This study confirmed that it is feasible to cultivate *C. cicadae* in the soil of a natural forest habitat, but the yield was lower than that of *C. cicadae* cultivated in sterile glass bottles. Our research also showed that different growth environments appear to affect the growth, bacterial community composition and metabolite content of *C. cicadae*, and bacterial community composition was correlated with metabolites content of *C. cicadae*.

Cultivating *C. cicadae* in natural habitats could help to meet consumer demand for natural products and reduce the exploitation of wild *C. cicadae* (Huang, 2008). However, in our study, the yield of *C. cicadae* cultivated in natural habitats was markedly reduced. Some research found that changes in growth environments can result in some resources being allocated to build a defense system to facilitate growth (Feng, 2008; Simon et al., 2010); it is therefore possible that, in the soil of their natural habitats, *C. cicadae* may also use resources to build a defense system to reduce the adverse effects of natural competitors and microorganisms on their growth. This would leave fewer resources allocated to growth of *C. cicadae*, which could have resulted in the reduced yield we observed in our study.

All organisms lived in complex microbial ecosystems and formed a close relationship with diverse microorganisms; changes in the growth environment can affect the microbial community associated with the host (Mueller et al., 2020). In this study, different growth environments were shown to affect bacterial community composition and dominant bacterial genera in the sclerotia of *C. cicadae*. *Pseudomonas* was significantly enriched in sclerotia of *C. cicadae* cultivated in the soil of a natural habitat. Researches showed that *Pseudomonas* was abundant in soil (Li et al., 2013), and Mou et al. (2021) found that *Pseudomonas* was the dominant genus in the sclerotia and external mycelial cortices of wild *C. cicadae*. We thought therefore the *Pseudomonas* in *C. cicadae* cultivated in a natural habitat may originated from the soil; if this is the case, this indicates that *C. cicadae* may communicate with microorganisms in the soil within its natural habitat. *Pseudomonas* spp., regarded as broad-host-range entomopathogenic bacteria, are known to exhibit insecticidal activity toward certain agricultural pests (Chen W.J. et al., 2014). This could potentially benefit the growth of *C. cicadae*, for example by promoting the mummification process, as previously observed in Chinese *Cordyceps* (Wu et al., 2020). In addition, in this study, the sclerotia of *C. cicadae* cultivated in glass bottles was rich in *Cedecea* bacteria, but this genus had a very low relative abundance in sclerotia of *C. cicadae* cultivated in natural habitats. Previous researches isolated and detected *Cedecea* in *C. cicadae* (Qu et al., 2019a, b); however, another study did not detect any *Cedecea* in sclerotia of *C. cicadae* (Mou et al., 2021), which indicated the abundance of *Cedecea* in *C. cicadae* is unstable and may be affected by the growth environment. Furthermore, in our study, bacterial community composition varied with the time point (and therefore growth stage) of *C. cicadae*, similar to previously published research (Wu et al., 2020). In particular, the relative abundance of *Enterococcus* gradually decreased with the growth of *C. cicadae*, suggesting that *I. cicadae* injected into the pupae may have inhibited some bacterial genera.

In this study, we found that the changes in relative abundance of bacteria OTUs related to some functions were consistent with changes in metabolite content: for example, the relative abundance of bacteria related to amino acid metabolism in *C. cicadae* in CS increased significantly than in CB, along with the content of total amino acids in this cultivation environment. The relative abundance of bacteria related to carbohydrate metabolism and glycan biosynthesis and metabolism in CS decreased significantly than in CB, along with the content of carbon compound (mannitol and polysaccharides) in this cultivation environment. Researches showed culture conditions (Cheng et al., 2012; Lee et al., 2019), nutrient sources (Mao et al., 2005; Chunyu et al., 2019), different strains (Kang et al., 2017), and hosts (He Y. et al., 2019) can affect the level of metabolites, but these factors were consistent between two cultivation environment in this study. As a result, the main reason for different level of metabolite in two cultivation environment may be caused by the difference of microbial community composition and related functions (Figures 5, 6). Studies on food fermentation also showed that metabolite production related closely to bacterial community composition in the progress of fermentation (He and Chung, 2020; Zang et al., 2020). Some studies also reported that endophytic microbes in *Cordyceps* spp. may affect the production of metabolites (Zhang et al., 2010; Qu et al., 2019a) and microbial co-culture can increase significantly the level of some metabolites (Akone et al., 2016; Yu et al., 2019). Therefore, further researches on determining which microbial species involve in the production of cordycepin, polysaccharide, adenosine, mannitol or amino acid and then using beneficially synthetic community or fungal-bacterial co-culture to improve the level of metabolites in *C. cicadae* are necessary. The amino acid content of edible mushrooms was known to be correlated with freshness and sweetness (Mau et al., 2001), which may explain why consumers prefer edible mushrooms grown in a natural habitat. In addition, the nitrogen compounds content of *C. cicadae* in natural soil habitats increased, while the carbon compounds decreased, which may suggest that *C. cicadae* may exchange carbon and nitrogen elements in the soil, similar function to *Metarhizium robertsii* (Behie et al., 2017; Barelli et al., 2019). We therefore speculated *C. cicadae* may exchange carbon and nitrogen elements in soil via endophytic bacteria to affect the content of carbon and nitrogen compounds in *C. cicadae*, which may potentially result in adding a branch to the carbon and nitrogen cycle in the ecosystem, entomogenous fungi *C. cicadae* is involved in carbon and nitrogen cycling in the soil.

Compared with the contents of metabolites in wild *C. cicadae* reported in other studies (Ge et al., 2007; Zhang et al., 2017), the total amino acids, polysaccharides and mannitol contents of *C. cicadae* in our study were higher regardless of how they were cultivated. *C. cicadae* cultivated in a natural habitat could achieve microbial communication in soil and be favored by consumer, it therefore be an alternative to the wild. Given the apparent close relationship between bacterial community composition and metabolites of *C. cicadae*, we will further isolate, test and screen microorganisms in future studies to identify which species may be beneficial to the growth and metabolite production of cultivated *C. cicadae*, so that the yield and metabolites contents could be improved by beneficial interaction between microorganisms.

## Conclusion

Our study demonstrated for the first time to our knowledge that cultivating *C. cicadae* in natural habitat soil is feasible, but further studies are required to improve yield. This cultivation method appeared to increase nitrogenous compound (amino acid) and decrease carbonaceous compound (mannitol and polysaccharides) content, possibly through the interaction *of C. cicadae* with microorganisms in the soil. Metabolite production was found to be closely related to the bacterial community during *C. cicadae* growth; *C. cicadae* may therefore also participate in the carbon and nitrogen cycle in natural ecosystems.

## Data Availability Statement

The datasets presented in this study can be found in online repositories. The names of the repository/repositories and accession number(s) can be found below: https://www.ncbi.nlm.nih.gov/, PRJNA698306.

## Author Contributions

ZZ designed and performed the experiments, analyzed the data, and wrote the manuscript. DM designed and performed the experiments and analyzed the data. LL, WZ, and LD collected the samples and performed the experiments. XZ designed the experiments and wrote the manuscript. All authors have read and approved the submission of the manuscript.

## Conflict of Interest

The authors declare that the research was conducted in the absence of any commercial or financial relationships that could be construed as a potential conflict of interest.

## Footnotes

1 http://picrust.github.io/picrust/
