# Supplementary material for: Different Cultivation Environments Affect the Yield, Bacterial Community and Metabolites of Cordyceps cicadae
Source: Front Microbiol. 2021 May 11;12:669785. doi: 10.3389/fmicb.2021.669785 (PMC8144455; doi:10.3389/fmicb.2021.669785)

Supplementary Material

Supplementary Table 1. Habitat information of experimental *Cordyceps cicadae* cultivation site.

Location	Geographical coordinates	Elevation(m)	Vegetation type	Dominant shrub	Soil type	Soil pH
West Campus of Guizhou University	106.6552E, 26.4541N	1112	*Pinus massoniana* forest	*Camellia oleifera*	Yellow soil	5.53

Supplementary Table 2. Relative yield of *C. cicadae* between CB and CS (CB, in glass bottle, CS, cultivated in the soil of a natural forest habitat, the data of the proportion of pupae producing *C. cicadae* fruiting bodies and mean fresh weight come from Fig 2, relative yield= number of cicada × proportion of pupae producing *C. cicadae* fruiting bodies × mean fresh weight per *C. cicadae*).

Group	Number of cicada	Proportion of pupae producing *C. cicadae* fruiting bodies	Mean fresh weight per *C. cicadae* (g)	Relative yield(g)
CS	100	92%	5.81	534.52
CB	100	95%	6.79	645.05

Supplementary Table 3. Coverage index of bacterial communities in *C. cicadae* cultivated under different environments (SCB, sclerotia of *C. cicadae* cultivating in glass bottles; SCS, sclerotia of *C. cicadae* cultivating in the soil of a natural habitat) at 1, 2 and 3 weeks after inoculation (_1, _2 and _3, respectively). Different lowercase letters depict the significant difference at $p = 0.05$.

Sample	Coverage index
SCB_1	0.9997 ±0.0006 bc

SCB_2	0.9995 ±0.0031 bc
SCB_3	0.9996 ±0.0011 bc
SCS_2	0.9988 ±0.0046 a
SCS_3	0.9994 ±0.0023 b

Supplementary Figure 1. Shannon index rarefaction curve of bacterial communities in sclerotia of *Cordyceps cicadae* cultivated under different environments (SCB, sclerotia of *Cordyceps cicadae* cultivating in glass bottles; SCS, sclerotia of *Cordyceps cicadae* cultivating in the soil of a natural habitat) at 1, 2 and 3 weeks after inoculation (_1, _2 and _3, respectively).

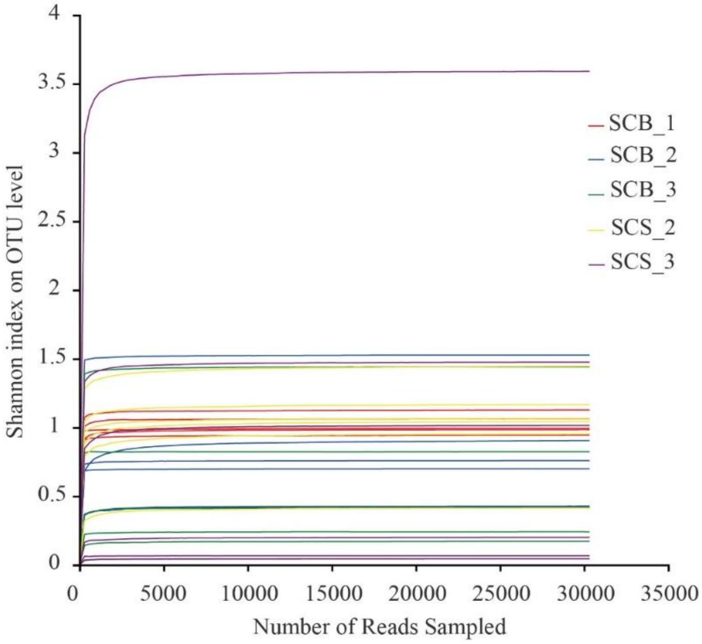

Supplement: Supplementary file 1 [file Data_Sheet_1.pdf]
